# Consumption of Roasted Coffee Leads to Conjugated Metabolites of Atractyligenin in Human Plasma

**DOI:** 10.1021/acs.jafc.3c05252

**Published:** 2023-11-30

**Authors:** Roman Lang, Coline Czech, Melanie Haas, Thomas Skurk

**Affiliations:** † Leibniz Institute for Food Systems Biology at the Technical University of Munich, Lise-Meitner-Str. 34, 85354 Freising, Germany; ‡ ZIEL—Institute for Food & Health, Core Facility Human Studies, Technical University of Munich, Gregor-Mendel-Str. 2, 85354 Freising, Germany

**Keywords:** roasted coffee, atractyligenin, metabolites, quantitative analysis, human intervention, plasma

## Abstract

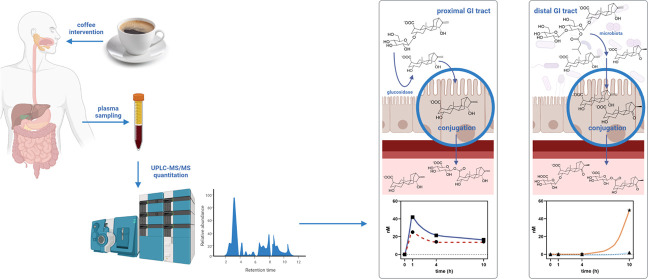

Roasted coffee contains atractyligenin-2-*O*-β-d-glucoside and 3′-*O*-β-d-glucosyl-2′-*O*-isovaleryl-2-*O*-β-d-glucosylatractyligenin, which are ingested with the brew. Known metabolites are atractyligenin, atractyligenin-19-*O*-β-d-glucuronide (**M1**), 2β-hydroxy-15-oxoatractylan-4α-carboxy-19-*O*-β-d-glucuronide (**M2**), and 2β-hydroxy-15-oxoatractylan-4α-carboxylic acid-2-*O*-β-d-glucuronide (**M3**), but the appearance and pharmacokinetic properties are unknown. Therefore, first time-resolved quantitative data of atractyligenin glycosides and their metabolites in plasma samples from a pilot human intervention study (*n* = 10) were acquired. None of the compounds were found in the control samples and before coffee consumption (*t* = 0 h). After coffee, neither of the atractyligenin glycosides appeared in the plasma, but the aglycone atractyligenin and the conjugated metabolite **M1** reached an estimated *c*_max_ of 41.9 ± 12.5 and 25.1 ± 4.9 nM, respectively, after 1 h. **M2** and **M3** were not quantifiable until their concentration enormously increased ≥4 h after coffee consumption, reaching an estimated *c*_max_ of 2.5 ± 1.9 and 55.0 ± 57.7 nM at *t* = 10 h. The data suggest that metabolites of atractyligenin could be exploited to indicate coffee consumption.

## 1 Introduction

**Figure 1:**
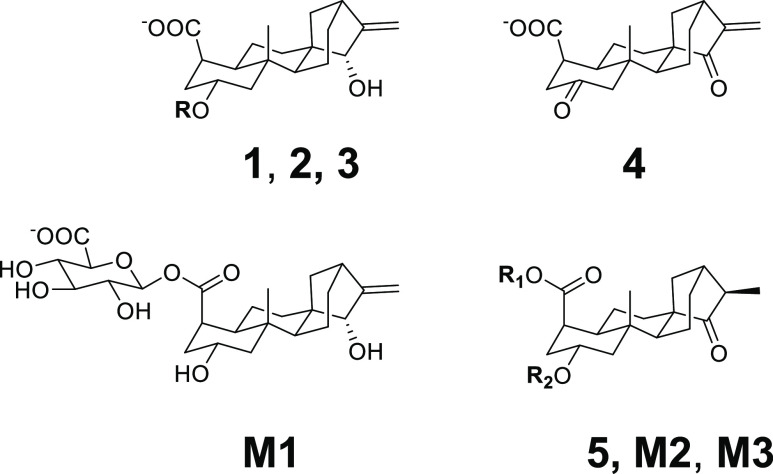
Structures of the analytes **1**–**3** and **M1**–**M3** and the internal standard (**4**). Atractyligenin (**1**, R = H), atractyligenin-2-*O*-β-d-glucoside (**2**, R = -β-d-glucose), 3′-*O*-β-d-glucosyl-2′-*O*-isovaleryl-2-*O*-β-d-glucosylatractyligenin (**3**, R = 3′-*O*-β-d-glucosyl-2′-*O*-isovaleryl-β-d-glucose). Internal standard (IS) 2,15-diketoatractyligenin (**4**). The aglycone of atractyligenin metabolites **M2** and **M3** is 2β-hydroxy-15-oxoatractylan-4α-carboxylic acid (**5**, R_1_, R_2_ = H). Conjugated metabolites isolated from urine: atractyligenin-19-*O*-β-d-glucuronide (**M1**), 2β-hydroxy-15-oxoatractylan-4α-carboxy-19-O-β-d-glucuronide (**M2**, R_1_ = -*O*-β-d-glucuronide, R_2_ = H), and 2β-hydroxy-15-oxoatractylan-4α-carboxylic acid-2-*O*-β-d-glucuronide (**M3**, R_1_ = H, R_2_ = -*O*-β-d-glucuronide).

Many people worldwide enjoy roasted coffee for its stimulating effects, for social reasons, or to regain concentration. Furthermore, coffee consumption is associated with a reduced risk of type 2 diabetes, possibly related to the abundant bioactive compounds of roasted coffee, e.g., caffeine, antioxidants, and melanoidins.^1−4^ Glycosides of the ent-kaurene atractyligenin (**1** in Figure 1) have been a compound class known in coffee since the first works from Obermann and Spiteller.^5−7^ Atractyligenin-2-*O*-β-d-glucoside (**2**) and 3′-*O*-β-d-glucosyl-2′-*O*-isovaleryl-2-*O*-β-d-glucosylatractyligenin (**3**) are the predominant derivatives. While their biological function for the coffee plant is unknown, their abundance is exceptionally high in Arabica coffee. It is around 0.8–1.4 mg/g (**2**) and 0.4–0.9 mg/g (**3**), respectively, while the aglycone itself (**1**) occurs only in traces.^8−10^ While glycosides of **1** have been reported in various plants, e.g., *Atractylis gummifera* L. or *Xanthium strumarium* L.,^11^ coffee is the only known food containing them in substantial amounts. This is supported by data from untargeted liquid chromatography–mass spectrometric (LC-MS) metabolomics studies with urine from coffee and noncoffee drinkers.^12^ Rothwell et al. suggested “atractyligenin glucuronide” as a potential biomarker for food intake (BFI) for coffee consumption. Shi et al. mentioned the detection of “atractyligenin glucuronide” in human plasma after coffee consumption.^13^ As to the underlying exact compound, Obermann et al. clarified the aglycone structure of an atractyligenin-related isobaric metabolite that was excreted into the urine after coffee consumption.^5^ This aglycone (**5** in Figure 1) was present in two of three conjugated metabolites, which we recently isolated from coffee drinkers′ urine.^14^ Therefore, the biomarker candidate “atractyligenin glucuronide” Rothwell et al. and Shi et al. referred to comprised the 19-*O*-β-d-glucuronide of **1**, termed **M1**, and the 19-*O*-β-d-glucuronide (**M2**) and 2-*O*-β-d-glucuronide (**M3**) of **5**.

Atractyligenin glycosides are extracted from the coffee powder into the beverage during coffee brewing; metabolites appear in plasma and are excreted after coffee consumption with the urine. It is yet unclear whether the glycosides **2** and **3** are bioavailable, and when and how metabolites are formed. Atractyligenin, its glycosides and metabolites are easily ionized due to the carboxyl functions at C19 and C6′, respectively, making ultra-performance liquid chromatography (UPLC) separation coupled to mass spectrometry the method of choice for selective and sensitive collection of targeted quantitative data.^10,14^ Therefore, the aim of the current investigation was to develop a targeted UPLC-MS/MS method, acquire first time-resolved quantitative data of atractyligenin metabolites in plasma samples from a pilot human coffee intervention study, and discuss their generation to address this question.

## 2 Materials and Methods

### 2.1 Chemicals

Chemicals were purchased from Sigma-Aldrich (Taufkirchen, Germany). Blank plasma was obtained from Hölzel Diagnostika Handels GmbH (Köln, Germany). Deuterated methanol for NMR was from Euriso-Top (Giv sur Ivette, France), and solvents for LC were from JT Baker (Deventer, The Netherlands). Water was taken from an Advantage A 10 System (Millipore, Molsheim, France). Atractyligenin (**1**) was a generous gift from M. Bruno (Department of Biological, Chemical and Pharmaceutical Sciences and Technologies, University of Palermo, 90128 Palermo, Italy). Small amounts of atractyligenin-2-*O*-β-d-glucoside (**2**) and 3′-*O*-β-d-glucosyl-2′-*O*-isovaleryl-2-*O*-β-d-glucosylatractyligenin (**3**) isolated from coffee (100% Arabica, Colombia) were available from previous studies.^10,14^ 2,15-diketoatractyligenin (**4**) was prepared by oxidation of **1** with Dess–Martin periodinane and subsequent purification by high-performance liquid chromatography (HPLC).^10,15^ Methanolic solutions of atractyligenin-19-*O*-β-d-glucuronide (**M1**), 2β-hydroxy-15-oxoatractylan-4α-carboxy-19-*O*-β-d-glucuronide (**M2**), and 2β-hydroxy-15-oxoatractylan-4α-carboxylic acid-2-*O*-β-d-glucuronide (**M3**) isolated from coffee drinkers’ urine and synthetic 2β-hydroxy-15-oxoatractylan-4α-carboxylic acid (**5**, 2.16 μmol/mL) were available from previous studies.^14^ Quantitative ^1^H NMR was done on a Bruker AV III system (Bruker, Rheinstetten, Germany) at 400 MHz, as reported.^16^ Acquisition of exact mass data (UPLC-time-of-flight MS) was done using a Shimadzu Nexera X2 UPLC system (Shimadzu, Duisburg, Germany) connected to a 6600 Triple ToF (Sciex, Darmstadt, Germany) as reported.^14^

### 2.2 Human Intervention Study

The study included ten volunteers (5 females, 5 males) (age: 28.3 ± 2.3 years; BMI: 21.89 ± 2.22 kg/m^2^). The intervention study took place from November 5, 2021, to February 17, 2022, in the core facility human studies of the ZIEL Institute for Food and Health of the Technical University of Munich. The study was performed in agreement with the Declaration of Helsinki, and all participants gave written informed consent. The study was approved by the Ethics Committee of the Technical University of Munich (5798/13 S-SR) and registered in the German Register of Clinical Studies (DRKS00005083).

The study participants underwent a dietary protocol that excluded all forms of coffee consumption. Additionally, chocolate, black/green/white, mate tea, cola, energy drinks, and beauty products containing caffeine (e.g., shampoos, creams) were prohibited 1 week before the test day. Furthermore, the study participants received a standardized meal the evening before the test day. The meal was composed of pasta, margarine, and salt, and the amount of energy was calculated based on the participant’s basal energy demand. On the morning of the test day, the participants were in a fastened state and received water (control samples) or coffee brew (coffee intervention) as breakfast. Blood was sampled before coffee intake (0 h) and at defined time points (1, 4, and 10 h) after coffee intake. 6 h after the coffee intake, the participants received lunch, the same standardized meal as the previous dinner. The administered coffee was prepared from a coffee capsule (Nespresso, ROMA) with 100 mL water. The administered volume of the coffee brew was 200 mL. Based on the coffee powder per capsule (6 g), the concentrations of **2** (2.8 μmol/g) and **3** (1.1 μmol/g), respectively,^10^ and an estimated extraction rate of 100%, the total ingested amounts were 33.6 μmol (**2**) and 13.2 μmol (**3**).

### 2.3 Stock Solutions

#### 2.3.1 Stock Solutions of **1**–**3** and the Internal Standard **4**

Solid atractyligenin (**1**, 1.44 mg, 4.5 μmol), atractyligenin-2-*O*-β-d-glucoside (**2**, 1.27 mg, 2.63 μmol), and 3′-*O*-β-d-glucosyl-2′-*O*-isovaleryl-2-*O*-β-d-glucosylatractyligenin (**3**, 1.06 mg, 1.46 μmol) were individually dissolved in ethanol (1 mL) as stock solutions. Aliquots of **1**–**3** individual stock solutions were combined and diluted with 20% aqueous ethanol to yield a working solution with 10 μM per compound. 2,15-Diketoatractyligenin (**4**) obtained by HPLC purification from synthesis (cf. Supporting Information) was dissolved in *d*_4_-methanol, and the concentration was determined by quantitative ^1^H NMR.^16^ The obtained solution was diluted appropriately with ethanol to 1000 nM final concentration in a measured flask (100 mL) and aliquoted to serve as the internal standard (IS) solution. The aglycone **5** was calibrated to calculate the concentration of the stock solutions **M2** and **M3** (cf. Supporting Information).

#### 2.3.2 Stock Solutions of **M1**–**M3**

The exact concentrations of the individual solutions of **M1**–**M3** were determined by enzymatic hydrolysis and quantification of the respective aglycone (cf. Supporting Information). The concentrations were 71.1 ± 4.8 nmol/mL (**M1**), 46.5 ± 3.5 nmol/mL (**M2**), and 13.9 ± 1.0 nmol/mL (**M3**). Aliquots were then combined and diluted with ethanol to yield a working solution with 10 μM (**M1**) and 5 μM (**M2** and **M3**).

### 2.4 Calibration Curves and Quality Controls

#### 2.4.1 Matrix Calibration Curves for **1**–**3**

The working solution of **1**–**3** was serially diluted in 1 + 1 steps with water to yield concentrations from 10,000 to 9.8 nM. Aliquots (100 μL) of the dilutions (10,000–9.8 nM) were spiked into blank plasma (900 μL) to yield matrix standards in the range from 1000 to 1 nM. Data from the UPLC-MS/MS analysis were used to calculate calibration curves by plotting area ratios of analyte to internal standard versus concentration ratios of analyte to internal standard. Quality controls (QCs) were prepared by spiking blank plasma (900 μL) with the **1**–**3** working solution in two concentrations. QCs were analyzed in replicates (*n* = 5).

#### 2.4.2 Matrix Calibration Curves for **M1**–**M3**

The working solution of **M1**–**M3** was serially diluted (1 + 1 steps) with water to 5000, 2500, 1250, 625, 312.5, 156.3, 78.1, 39.1, 19.5, and 9.8 nM. Aliquots (100 μL) were mixed with blank plasma (900 μL) to yield matrix standards spiked from 500 to 1 nM (**M1**) and 250 to 0.5 nM (**M2**, **M3**). Data from the UPLC-MS/MS analysis were used to calculate the calibration curves by plotting area ratios of the analyte to internal standard versus concentration ratios of the analyte to internal standard. Quality controls (QCs) were prepared by spiking blank plasma (900 μL) with the **1**–**3** working solution in two concentrations. QCs were analyzed in replicates (*n* = 5).

### 2.5 Sample Preparation

In an Eppendorf tube (1.5 mL), aliquots (50 μL each) of the calibration standard, sample, or QC, respectively, were mixed with the IS solution (1000 nM in ethanol, 50 μL) and acetonitrile/ethanol (9 + 1, v + v, 50 μL) was added. After vortexing, suspensions were cleared by centrifugation (12,000 rpm, 4 °C, 10 min), and the supernatant (∼100 μL) was transferred to an HPLC vial with 200 μL insert for analysis (5 μL injection).

### 2.6 Quantitative Analysis

#### 2.6.1 Instrumentation

The UPLC–MS/MS system consisted of a Shimadzu Nexera X2 UPLC system (Shimadzu, Duisburg, Germany) hyphenated to a QTrap 5500 MS/MS system (Sciex, Darmstadt, Germany) operating in negative electrospray (ESI^–^) mode with the following ion source parameters: ion spray voltage: −4500 V; source temperature: 500 °C; nebulizer gas: 55 psi; heater gas: 65 psi; and curtain gas: 35 psi. The MS/MS parameters, including the collision cell entrance potential (CEP), declustering potential (DP), collision energy (CE), and cell exit potential (CXP), were tuned for each compound and mass transition and are summarized in Supporting Table S3. The dwell time for each compound was 30 ms, and the total cycle time was 0.75 s. Chromatographic separation was achieved on a C18 column (Kinetex C18, 100 mm × 2.1 mm, 1.7 μm, Phenomenex, Aschaffenburg, Germany) at a flow rate of 500 μL/min flow rate. Eluent A was 0.1% formic acid in water, and eluent B was 0.1% formic acid in acetonitrile. B was kept at 3% (1 min), increased to 25% (in 3 min), 90% (in 2 min, 1 min isocratic), and 3% (0.5 min, 2.5 min isocratic). The total time of the analysis was 10 min. The column effluent was introduced from the waste to the MS/MS system between 3 and 7 min.

### 2.7 Calculations and Illustrations

Calibration curves, precision and accuracy, and concentrations were calculated in Analyst 1.6.3 and Microsoft Excel 2016. Data analysis and visualization were done in GraphPad Prism 9.3.0 for Windows (GraphPad Software, San Diego, California, www.graphpad.com). Illustrations (TOC graphic and Figure 3) were created with Biorender.com.

## 3 Results

Roasted coffee contains the glycosides atractyligenin-2-*O*-β-d-glucoside and 3′-*O*-β-d-glucosyl-2′-*O*-isovaleryl-2-*O*-β-d-glucosylatractyligenin (**2** and **3** in Figure 1). In our recent studies, we isolated three conjugated metabolites (**M1**–**M3** in Figure 1) of dietary atractyligenin glycosides from coffee drinkers’ urine and clarified the structures.^14^ In the current study, we quantitatively investigated the appearance of the original compounds **2** and **3** from coffee and their metabolites **1** and **M1**–**M3** in plasma samples from a coffee intervention study.

We used the isolated conjugates and optimized the ion path and fragmentation parameters during method development to allow detection in targeted UPLC-MS/MS analysis. Tuning involved individual infusion of **M1**–**M3** and software-assisted optimization of the ion source parameters in negative electrospray (ESI^–^), leading to intense pseudomolecular ions *m*/*z* 495.2 ([M – H]^−^). Collision-induced dissociation led to fragments *m*/*z* 319.2 ([M-glucuronic acid-H]^−^), *m*/*z* 192.9 ([M-aglycone-H^–^]), and *m*/*z* 174.9 ([M-aglycone-H_2_O–H^–^]), indicating cleavage of aglycone and connected glucuronic acid (cf. Supporting Figures S4 – S6), and three mass transitions per compound were recorded. The tunings were combined with the individual tunings of the coffee compounds **1**–**3** and the synthetic derivative 2,15-diketoatractyligenin (**4**, cf.^10^), which we used as the internal standard for quantitation. The isobaric metabolites **M1**–**M3**, the original compounds **1**–**3** present in roasted coffee, and the IS (**4**, cf.^17^) were successfully separated by UPLC on C18 within 10 min with excellent retention time repeatability (Table 1 and Supporting Figure S2). We further included compound **5**, which is the aglycone of metabolites **M2** and **M3** and isobaric to **1**, into the method to determine the concentration of the isolates from coffee drinkers’ urine in the respective stock solution (cf. Supporting Information).

**Table 1:** Chromatographic and Spectrometric Parameters of the Analytes **1**–**3** and **M1**–**M3**

analyte	RT (min)^a^	Q1/Q3 (*m*/*z*)^b^	calibrated range (*R*^2^)^c^	LLOQ (nM)^d^	precision (%)^e^	accuracy (%)^e^
1	5.02 (±0.01)	319.1/275.2*, 273.2	15.6 – 500.0 (0.993)	15.6 (S/N ≥ 31)	2.7–17.7	96.3–104.7
2	4.38 (±0.01)	481.2/118.9, 59.1*	31.3 – 500.0 (0.990)	31.3 (S/N ≥ 36)	4.4–24.3	86.1–107.8
3	5.21 (±0.01)	727.3/643.3*, 625.4	2.0 – 500.0 (0.998)	2.0 (S/N ≥ 23)	1.0–18.9	96.4–104.2
M1	4.38 (±0.01)	495.2/319.2*, 192.9, 174.8	2.0 – 250.0 (0.997)	2.0 (S/N ≥ 39)	2.0–16.1	83.7–109.5
M2	4.88 (±0.01)	495.2/319.2*, 174.9, 113.0, 84.9	0.5 – 62.5 (0.996)	0.5 (S/N ≥ 18)	2.6–24.7	85.2–106.5
M3	5.09 (±0.01)	495.2/319.1, 192.9*, 113.0, 59.0	1.0 – 62.5 (0.994)	1.0 (S/N ≥ 15)	2.3–18.1	88.6–108.7
4 (IS)	5.46 (±0.01)	315.1/271.2, 253.2*				

a Calculated from plasma analyses and plasma calibration standards.

b Quantifier is marked w/asterisk.

c Calibrated range with precision <25% at LLOQ and accuracy 80–120%.

d LLOQ: lower limit of quantitation defined as the smallest concentration in the calibrated range.

e Values from back-calculated calibration standards in the plasma matrix (cf. Supporting Information).

We prepared calibration standards for **1**–**3** and **M1**–**M3** in the blank matrix to quantify the compounds in samples of human plasma taken after water (control) and coffee intervention. Sample preparation involved the addition of the internal standard to the sample and the precipitation of plasma proteins using acetonitrile. Supernatants obtained after centrifugation were directly injected. Qualifier and quantifier, retention time, calibrated range in the plasma matrix, precision (as the relative standard deviation of replicates) and accuracy values for back-calculated calibration standards are summarized in Table 1 and Supporting Table S4. Results from analyses of quality control samples (QCs) prepared in blank plasma are listed in Table 2. The conjugates **M1**–**M3** formed intense product ions with a good signal/noise ratio. QCs indicated good precision ≤9.6% (relative standard deviation, RSD) at the low concentrated QC and ≤7.0% at the highly concentrated QC. Recovery was 103.9–112.4% at the low QC and 96.1–106.9% at the high QC. The lower limit of quantification (LLOQ) of compounds **1** and **2** was 15.6 and 31.3 nM, and that of compound **3** was 2 nM. Precision was ≤15.9% at the low QC and ≤6.8% at the high QC. Accuracy ranged between 77.9 and 112.4% at the low QC and 83.8 and 96.1% at the high QC.

**Table 2:** Quality Controls

	high QC				low QC			
analyte	nom. (nM)	found (nM)	RSD (%)	accuracy (%)	nom. (nM)	found (nM)	RSD (%)	accuracy (%)
1	500.0	480.4 (±17.3)	3.6	96.1	15.6	17.5 (±2.6)	14.7	112.4
2	500.0	444.8 (±24.5)	5.5	88.9	15.6	12.2 (±1.9)^a^	15.9	77.9
3	500.0	419.1 (±28.2)	6.8	83.8	15.6	13.7 (±1.0)	7.3	87.9
M1	125.0	133.3 (±9.4)	7.0	106.6	7.8	8.3 (±0.8)	9.6	106.1
M2	62.5	65.7 (±4.6)	7.0	105.2	3.9	4.1 (±0.2)	4.6	103.9
M3	62.5	66.9 (±4.7)	6.9	106.9	3.9	4.4 (±0.3)	7.1	112.2

a Below LLOQ; data are means ± standard deviation of replicates (*n* = 5).

We applied this method to human plasma samples from a coffee intervention study to investigate the appearance of coffee compounds **1**–**3** and metabolites **M1**–**M3**. Ten participants underwent a washout period (7 days) and then consumed one dose of tab water. Blood samples were taken at baseline (0 h) and 1, 4, and 10 h after the intervention. The same individuals underwent the identical procedure but consumed one dose of roasted coffee brew (200 mL) prepared from two coffee capsules. Again, samples were taken at baseline (0 h) and 1, 4, and 10 h after the coffee intake.

**Figure 2:**
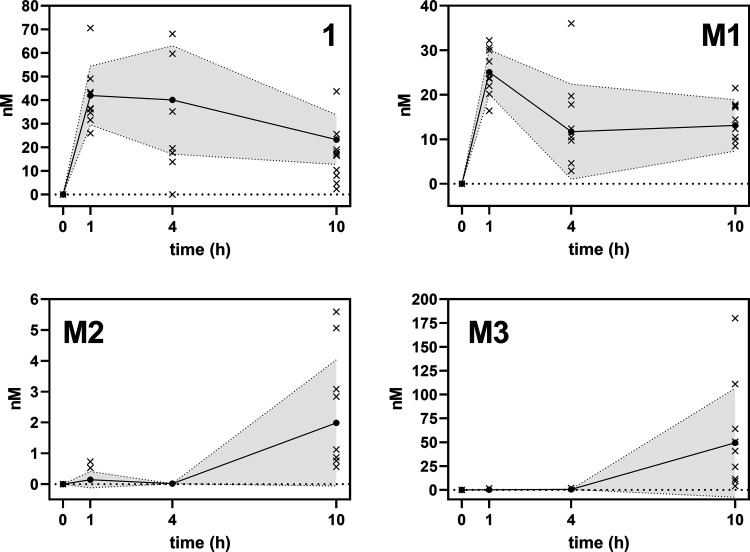
Concentration–time (nM × h) profiles of atractyligenin (**1**) and the conjugated metabolites **M1**–**M3** in plasma after coffee brew (200 mL). Figures contain individual values (×, *n* = 10 study participants) and means ± SD (●, gray area). Cf. Supporting Table S5 for tabulated individual concentrations.

Analysis of the plasma samples of the control group who had ingested a dose of tab water resulted in the complete absence of the coffee compounds **1**–**3** and the metabolites **M1**–**M3**, indicating good compliance with the study protocol. When 200 mL of roasted coffee brew was consumed, **1** and **M1**–**M3** were quantifiable in the samples. However, the concentration was relatively low. (Supporting Table S5 gives the individually calculated concentrations. Concentrations below the lower limit of quantitation (LLOQ) are marked with an asterisk.) The data are summarized in Figure 2.

None of the samples contained the atractyligenin glycosides **2** and **3** from coffee above the LLOQ. In contrast, atractyligenin (**1**) was detected in every individual 1 h after coffee consumption (41.9 ± 12.5 nM). After 4 h, **1** was still present above the LLOQ in five individuals (40.1 ± 22.9 nM) and after 10 h in six individuals (23.3 ± 10.5 nM). For **M1**, no peaks were detected at the baseline. Upon coffee ingestion, **M1** reached its estimated maximum *c*_max_ (25.1 ± 4.9 nM) after 1 h. The concentration was significantly lower after 4 h (14.2 ± 10.5 nM) in 80% of the samples above the LLOQ. The concentration then remained stable, as even after 10 h still, 14.4 ± 4.4 nM (90% above the LLOQ) was found. **M1** was above the LLOQ in almost every sample, underlining our previous assumption that it constitutes the primary conjugated metabolite of dietary atractyligenin glycosides.^14^**M2** was below the LLOQ at the baseline and even in most samples taken after coffee consumption. After 1 h, **M2** was above the LLOQ in only two of the ten participants (concentration 0.63 ± 0.14 nM). However, after 10 h, it was found in 80% of the samples and reached a maximum concentration *C*_max_ of 2.5 ± 1.9 nM. **M3** was below the LLOQ at the baseline. After 1 h, traces were found in one individual. After 10 h, it was above the LLOQ in 90% of the samples and reached a maximum concentration *C*_max_ of 55.0 ± 57.7 nM. Interestingly, comparatively high concentrations of 111.0 and 180.0 nM, after 10 h, respectively, were found in the samples from two individuals.

## 4 Discussion

For this paper, we applied a newly developed targeted UPLC-MS/MS method to quantify the metabolites of dietary atractyligenin glycosides (Figure 1) in human plasma samples from a coffee intervention study to discuss the potential use of the metabolites as biomarkers for food intake (BFI) for coffee consumption.

Atractyligenin glycosides are water-soluble and abundant in Arabica coffee, which is a substantial part of coffee blends in many cases. While atractyligenin glycosides have been reported in several toxic plants and weeds,^11^ they are absent in foods other than coffee. This coffee specificity and their appearance in plasma and urine after coffee suggest that atractyligenin metabolites might be exploited as potential dietary biomarkers for coffee consumption.^12−14^ Therefore, the present investigation aimed to evaluate concentration–time profiles of atractyligenin derivatives from coffee and their metabolites in plasma after consumption of roasted coffee brew for the first time.

The study participants underwent a dietary protocol that excluded the use and consumption of every form of coffee preparation. As a result, no atractyligenin metabolites were found in plasma samples, indicating that roasted coffee was the only dietary or environmental source and thus specific for roasted coffee. This is in line with food chemistry data on the compound class and previous untargeted metabolomics data, which concluded that atractyligenin glucuronide was specific to coffee drinkers.^12−14^

**Figure 3:**
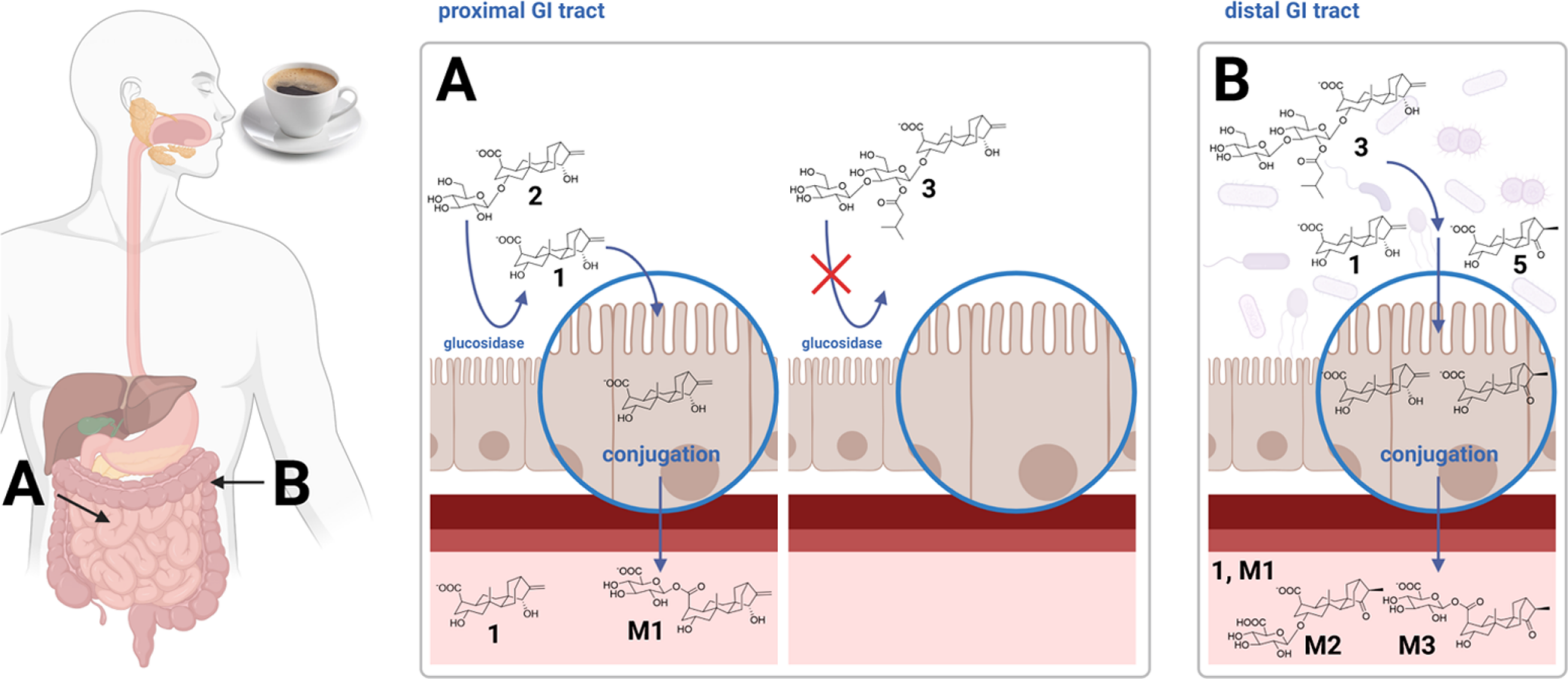
Coffee brew provides atractyligenin glycosides **2** and **3**. (A) The monoglucoside **2** from coffee might be cleaved into **1** by glucosidases in the proximal GI tract, followed by subsequent uptake of the aglycone, leading to plasma appearance of **1** and **M1**. The esterified diglycoside **3** from coffee might not be a substrate for glucosidases, so it remains intact and transfers into the distal GI tract (B), where gut microbiota removes the sugar moiety and metabolizes **3**, releasing **1** and **5**. **1** and **5** are taken up and appear as glucuronides in the plasma. This illustration was created with Biorender.com.

However, according to our data after coffee consumption, none of the atractyligenin glycosides abundant in roasted coffee was found, while aglycone atractyligenin (**1**) appeared already 1 h after coffee. As **1** is only a trace compound in roasted coffee,^8,10^ we assume that, in particular, **2** was enzymatically deglycosylated upon ingestion in the gut, thereby explaining the appearance of the aglycone **1** in plasma (Figure 3A). Németh et al. (2003) isolated β-glucosidases lactase phlorizin hydrolase (LPH) and cytosolic β-glucosidase (CBG) from the human small intestine.^18^ They report that the enzymes substantially contributed to the metabolism of dietary flavonoid glycosides, as they liberated the aglycone from the monoglycosidic form, facilitating its uptake into the cells (cf. the review by Murota et al.^19^). Despite the difference in compound class, atractyligenin-2-*O*–d-glucoside (**2**) possibly was a target for the glucosidases and similarly cleaved, leading to the early appearance of **1** in the plasma. Németh et al. further report that liberated aglycones can be taken up in the small intestine by, e.g., passive diffusion and conjugated in the epithelial cells by cytosolic enzymes to form phase II metabolites (Figure 3A), in addition to phase II metabolism in the liver.^18^ This observation could explain the early appearance of **M1**, a conjugated metabolite of structurally unaltered atractyligenin. The observation that the aglycone **1** and its glucuronide **M1** are both detectable in plasma after coffee differs from other orally administered dietary glycosides, e.g., quercetin-3′-*O*-glucoside or steviol glycosides.^20,21^

The respective panels in Figure 2 show that after reaching an estimated *C*_max_ after 1 h, the plasma concentration of **1** stays relatively stable between 1 and 4 h and drops by ∼45% until 10 h. We speculate that, in contrast to atractyligenin-2-*O*-β-d-glucoside (**2**), the coffee-derived glycoside **3** is not a substrate for LPH or CBG in the proximal GI tract (Figure 3A) but was cleaved by β-glucosidase activity of the intestinal microbiota in the large intestine.^22^ After early liberation of **1** by hydrolysis of **2** through epithelial glucosidases, the liberation of **1** from **3** by microbial glucosidases and subsequent uptake of the aglycone **1** could counteract the metabolism- and excretion-mediated plasma-clearance of **1**, explaining the prolonged plasma levels. However, the individual plots (Supporting Figure S3) indicate that after going through a first estimated maximum 1 h after coffee, the plasma concentrations of **1** and its 19-*O*-glucuronide **M1** increased between 4 and 10 h in 60% of the participants. This observation supports the assumption that **1** is liberated from **2** and **3** in two different digestive tract regions. Atractyligenin glycosides belong to the same compound class as steviol glycosides, e.g., stevioside. It is known that stevioside is not hydrolyzed by enzymes of the GI tract but by the colon microbiota.^21^ We thus speculate that diglycoside **3** is similarly hydrolyzed in the colon only.

While glucosidases in the small intestine might ignore atractyligenin glycoside **3** as a substrate, the microbiota is probably involved in both hydrolysis of the glycoside and, to some extent, structural modification of the aglycone from **1** to **5** (Figure 3B). Metabolite **M1** went through its estimated plasma maximum after 1 h and remained relatively constant between 4 and 10 h. We assume that hydrolysis of **3** by gut microbiota supplies **1** to form **M1**, stabilizing its plasma concentration.

In contrast to **M1**, conjugated metabolites **M2** and **M3** are the 19- and 2-*O*-β-d-glucuronides of compound **5**. **5** is an isobaric derivative of **1**, in which the exocyclic methylene group is reduced to the methyl group and the secondary alcohol at C15 is oxidized into the carbonyl. **5** was first reported by Obermann et al. as the aglycone of a conjugated atractyligenin metabolite and later confirmed by Lang et al.^5,14^ While **5** did not appear in the plasma, **M2** and **M3** steeply increased from barely quantifiable after 4 h to reach maximum values after 10 h. **M2**, however, showed minor concentrations, while the concentration of **M3** increased to finally exceed that of **1** and **M1** 10 h after coffee. We assume part of the atractyligenin glycosides, particularly **3**, were metabolized by the intestinal microbiota, leading to these late-appearing metabolites (Figure 3B).

While this is the first presentation of time-resolved quantitative data of atractyligenin metabolites in plasma after coffee intake, it is a limitation that only the four time points 0 h, and 1, 4, and 10 h after coffee intake were available, which only allows a rough estimation of peak plasma concentration. However, the data indicate that **1** and the conjugated metabolites **M1**–**M3** might be detectable for a considerable time in plasma. The individual concentration–time plots (Supporting Figure S3) imply individual parameters, e.g., the constitution of gut microbiota might contribute to their formation. Unlike metabolites **1** and **M1**, the conjugates **M2** and **M3** appeared in 80 and 90% of the study participants within 10 h after coffee. This picture is comparable to the qualitative data in urine reported in our recent paper,^14^ where we detected **1** and **M1** in every sample and **M2** and **M3** in four of six samples (67%).

BFI are suggested as tools for compliance control and to reduce misqualification in health-related nutritional studies.^23^ As they are parameters that can be collected by instrumental analysis, they are considered superior to food frequency questionnaires or interviews. While the full validation as a biomarker in terms of the proposed criteria (“plausibility”, “dose–response”, “time–response”, “robustness”, “reliability”, “stability”, “analytical performance”, and “reproducibility”) is beyond the purpose of this paper, the data provided here nevertheless support the suggestion that atractyligenin metabolites hold potential as BFI.^12,13^ Other compounds discussed as BFI candidates are metabolites of chlorogenic acids (CGAs) or caffeine. However, while coffee is the primary dietary source for CGAs, this compound class is abundant in many plant-based staple foods, e.g., potatoes, and therefore not specific to coffee.^3,24^ Similarly, coffee is the primary dietary source for the alkaloid caffeine, but methylxanthines are present in cocoa, black tea, energy drinks, and caffeinated sodas.^2^ In contrast, (Arabica-) coffee is the only food item that contains substantial amounts of atractyligenin glycosides. The structures of important metabolites are clarified,^5,12,14^ and the quantitative information provided in this paper indicates their appearance in plasma could be used to detect recent coffee consumption.

## Data Availability

All data are incorporated in the main manuscript and the Supporting Information File.

## Supporting Information Available

Synthesis of 2,15-diketoatractyligenin with MS and NMR data; determination of the concentration of isolated metabolites **M1**–**M3** (Figure S1 and Tables S1 and S2); ion path parameters of the MS/MS system (Table S3); MRM traces of the analytes in plasma (Figure S2); back-calculated concentrations, precision and accuracy of matrix calibration curves for **1**–**3** and **M1**–**M**3 in plasma (Figure S7 and Table S4); tabulated quantitative data in plasma samples from the coffee intervention study (Table S5); fragment spectra of **M1**–**M3** (Figures S4–S6) (PDF)

## Abbreviations

UPLC-MS/MS: ultra-performance liquid chromatography with tandem mass spectrometric detection
UPLC-time-of-flight MS: ultra-performance liquid chromatography with time-of-flight mass spectrometric detection
NMR: nuclear magnetic resonance spectroscopy
