# Supplementary material for: Consumption of Roasted Coffee Leads to Conjugated Metabolites of Atractyligenin in Human Plasma
Source: J Agric Food Chem. 2023 Nov 30;71(49):19516–22. doi: 10.1021/acs.jafc.3c05252 (PMC10722499; doi:10.1021/acs.jafc.3c05252)
Supplement: Supplementary file 1 — jf3c05252_si_001.pdf [file jf3c05252_si_001.pdf]

# Supporting Information

## Consumption of roasted coffee leads to conjugated metabolites of atractyligenin in human plasma

Roman Lang,[†,*] Coline Czech,[†] Melanie Haas,[‡] Thomas Skurk [‡]

[†] Leibniz Institute for Food Systems Biology at the Technical University of Munich, Lise-Meitner-Str. 34, 85354 Freising, Germany

[‡] ZIEL - Institute for Food & Health, Core Facility Human Studies, Technical University of Munich, Gregor-Mendel-Str. 2, 85354 Freising, Germany

*Corresponding author: Roman Lang, Email: r.lang.leibniz-lsb@tum.de, orcid.org/0000-0003-0610-7186; Phone: +49 (0) 8161-712978

### Supplemental information (SI)

The SI file contains the synthesis of 2,15-diketoatractyligenin with MS and NMR data; determination of the concentration of isolated metabolites M1 – M3 (Supporting Figure S1, Supporting Table S1, Supporting Table S2); Ion path parameters of the MS/MS system (Supporting Table S3); MRM traces of the analytes in plasma (Supporting Figure S2); back-calculated concentrations, precision and accuracy of matrix calibration curves for 1 – 3 and M1 – M3 in plasma (Supporting Figure S7, Supporting Table S4); tabulated quantitative data in plasma samples from the coffee intervention study (Supporting Table S5); fragment spectra of M1 – M3 (Supporting Figure S4, Supporting Figure S5, Supporting Figure S6).

*Synthesis of 2,15-diketoatractyligenin (**4**, IS).*

Atractyligenin (**2**, 15 mg, 46 µmol) was dissolved in acetone (8 ml), mixed with solid Dess-Martin-Periodinan (DMP, 20 mg) and incubated (48h, RT). The suspension was centrifuged, and the supernatant was again mixed with solid DMP (10 mg) and incubated (12h, RT). The suspension was centrifuged, and the supernatants were combined and evaporated. The residue was taken up in water (8 ml) and purified by semipreparative HPLC (202 nm) on a Hyperclone C18 column (250 mm×10

mm, 5 µm, Phenomenex, Aschaffenburg, Germany). Eluents were 0.1% formic acid in milliQ water (eluent A) and acetonitrile (eluent B); the flow rate was 5 ml/min. The binary gradient started with 5% B (isocratic for 2 min) and was increased to 100% within 23 min (isocratic for 5 min). 2,15-diketoatractyligenin eluted after 15.5 min. Peaks were collected manually and dried by lyophilization. The yield of purified 2,15-diketoatractyligenin was 5.32 µmol (11.5%).

NMR and MS data were in accordance with the literature (Lang et al., 2013). 2,15-diketoatractyligenin (**4**, **IS**). Retention time UPLC-ToF-MS (cf. method and instrumentation in Lang et al., 2022): 12.70 min, MS (ESI$^-$) found m/z 315.1609, calculated 315.1591 ($C_{19}H_{23}O_4$, $[M-H]^-$), Δ5.9 ppm.

$^1$H NMR ($d_4$-methanol): 1.05 (s, 3H, H20), 1.42 (m, H, H14), 1.43 (s, H, H9), 1.69 (m, 1H, H12), 1.85 (m, 1H, H11), 1.90 (tdd, J=13.1, 6.0, 2.6 Hz, 1H, H12), 1.98 (d, J=13.8 Hz, 1H, H1), 2.01 (m, 1H, H11), 2.16 (m, 1H, H5), 2.39 (d, J=12.1 Hz, H14), 2.52 (d, J=13.8 Hz, 1H, H1), 2.55 (m, 1H, H3), 2.75 (dt, 1H, H3), 3.08 (m, 1H, H13), 3.10 (m, 1H, H4), 5.35 (s, 1H, H17), 5.92 (s, 1H, H17); $^{13}$C NMR ($d_4$-methanol): 17.45 (C20), 19.20 (C11), 25.40 (C6), 32.86 (C12), 34.13 (C7), 37.04 (C14), 39.38 (C13), 44.11 (C3), 44.56 (C10), 46.49 (C4), 48.30 (C5), 51.69 (CH, C9), 53.24 (C, C8), 56.22 ($CH_2$, C1), 115.44 ($CH_2$, C17), 151.02 (C, C16), 177.71 (C, C19), 211.78 (C, C2), 211.94 (C, C15).

*Quantitative $^1$H NMR to determine the concentration*. The white residue of the evaporated 2,15-diketoatractylgenin was dissolved in $d_4$-methanol (2000 µl) to determine the concentration by quantitative $^1$H NMR (qNMR). Signals used to calculate the concentration were the exocyclic protons of C17 at δ 5.92 and 5.34 ppm. The concentration was determined to be 2.66 µmol/ml.

*Determination of the concentration of solutions of metabolites **M1** – **M3**.*

*Calibration curve for **1** and **5***. Aliquots of the 2β-hydroxy-15-oxoatractylan-4α-carboxylic acid (**5**, 2.16 µmol/ml) and atractyligenin (**1**, 4.50 µmol/ml) stock solution were combined and diluted with ethanol to 100 nmol/ml final concentration. This solution was diluted with water to 5000, 1000, 500, and 100 nM. Aliquots (100 µl) were mixed with the internal standard solution (2,15-diketoatractyligenin, 1000 nM, 100 µl) and analyzed by UPLC-MS/MS. The calibration curves (area ratios analyte/IS versus concentration) were used to calculate the concentration of **M1** – **M3** based on the concentration of the enzymatically liberated aglycone.

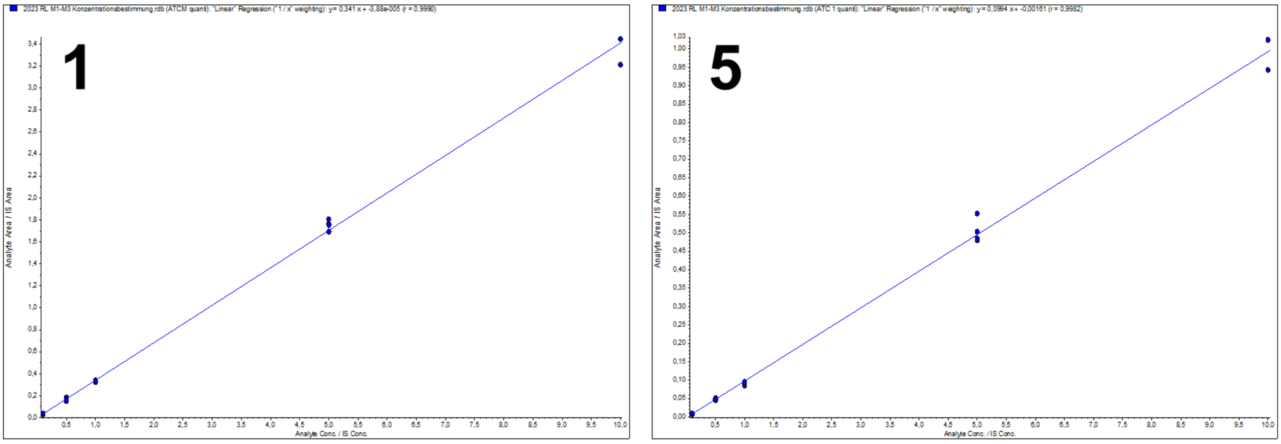


***Figure S1***. *Calibration curves of atractyligenin (**1**) and 2β-hydroxy-15-oxoatractylan-4α-carboxylic acid (**5**) were used for the determination of the concentration of the solution of conjugated metabolites **M1** – **M3**.*

***Table S1***. *Precision and accuracy of the calibration curves used to determine the concentration of **M1** - **M3**.*

| **Nom.Conc. (nM)** | **1** | | | **5** | | |
|---|---|---|---|---|---|---|
| | Found (nM) | RSD (%) | Accuracy (%) | Found (nM) | RSD (%) | Accuracy (%) |
| 100 | 102±10 | 9.8 | 102.6 | 104±11 | 10.6 | 104.9 |
| 500 | 490±45 | 9.2 | 97.9 | 503±29 | 5.9 | 100.7 |
| 1000 | 972±23 | 2.4 | 97.2 | 924±50 | 5.5 | 92.4 |
| 5000 | 5144±137 | 2.7 | 102.9 | 5095±333 | 6.6 | 101.9 |
| 10000 | 9764±487 | 4.9 | 97.6 | 9909±581 | 5.9 | 99.9 |

Data are means of n=2-4

*Hydrolysis of **M1** – **M3***. In Eppendorf Caps, aliquots of the solutions of **M1** – **M3** isolated from coffee drinkers´ urine (20 µl) were individually mixed with aqueous sodium acetate (3 g/100 ml, pH 4.8, 180 µl) and β-glucuronidase (20 µl), and incubated with shaking (12h, 37°C). When no intact conjugated metabolite was detectable in UPLC-MS/MS, an aliquot (20µl) was diluted and mixed with 2,15-diketoatractyligenin (1000 nM in ethanol, 20µl) and acetonitrile/ethanol (9+1,v+v). After centrifugation (5 min, 12000 rpm), the supernatant was analyzed by UPLC-MS/MS. The concentration of the liberated aglycone, corresponding to the respective intact conjugated metabolite, was calculated using the calibration curve. Each conjugated analyte was hydrolyzed in triplicates. The concentrations used for further preparation of matrix calibration curves were 71.1 nmol/ml (**M1**), 46.5 nmol/ml (**M2**), and 13.9 nmol/ml (**M3**).

***Table S2**. Results of the determination of the concentration of **M1 – M3**.*

| | M1 | M2 | M3 |
|---|---|---|---|
| Aglycone | 1 | 5 | 5 |
| Found (nmol/ml) | 71.1±4.7 | 46.5±3.5 | 13.9±1.0 |
| RSD, % | 6.7 | 7.6 | 7.3 |

Data are means of n=3

***Table S3**. ion path parameters for MS/MS detection.*

| analyte | Q1/Q3 (m/z) [a] | DP | CE | CXP |
|---|---|---|---|---|
| 1 | 319.09/275.2*, 273.2 | -45 | -34, -36 | -5, -3 |
| 2 | 481.18/118.9, 59.1* | -5 | -76, -36 | -7, -11 |
| 3 | 727.26/643.3*, 625.4 | -15 | -46, -46 | -9, -1 |
| M1 | 495.16/319.2*, 192.9, 174.8 | -30 | -36, -26, -30 | -3, -13, -13 |
| M2 | 495.17/319.2*, 174.9, 113.0, 84.9 | -20 | -36, -28, -30 | -5, -13, -3 |
| M3 | 495.16/319.1, 192.9*, 113.0, 59.0 | -10 | -38, -44, -44 | -5, .9, -11 |
| 4 (IS) | 315.07/271.2, 253.2* | -40 | -28, -32 | -3, -7 |
| 5 | 319.09/275.2*, 273.2 | -45 | -38, -36 | -7, -5 |

Entrance potential (EP) was -10 V for every analyte. Dwell time was 10 msec for each analyte.

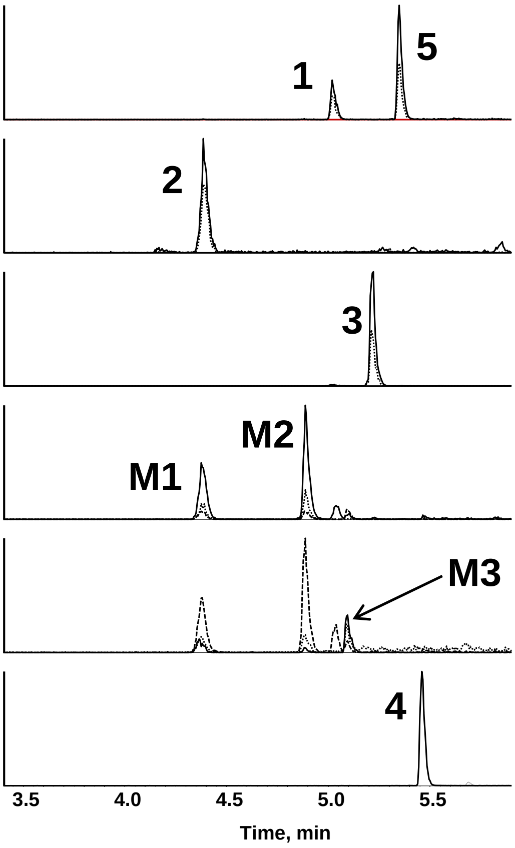


***Figure S2**. Multiple Reaction Monitoring (MRM) traces of the coffee compounds **1 – 3**, compound **5**, conjugated metabolites **M1 – M3** isolated from coffee drinkers´ urine, and the internal standard **4** in blank plasma. Quantifier traces are solid; qualifiers are dotted and dashed (**Table 1**).*

***Table S4**. Back-calculated values of matrix calibration standards (**1 – 3**, **M1 – M3**) in blank plasma.*

| **Nom. (nM)** | **1** | | | **2** | | | **3** | | |
|---|---|---|---|---|---|---|---|---|---|
| | Found (nM) | RSD (%) | Accuracy (%) | Found (nM) | RSD (%) | Accuracy (%) | Found (nM) | RSD (%) | Accuracy (%) |
| 0.5 | | | | | | | | | |
| 1.0 | | | | | | | | | |
| 2.0 | | | | | | | 1.9±0.4 | 18.9 | 98.7 |
| 3.9 | | | | | | | 3.8±0.1 | 2.9 | 98.3 |
| 7.8 | | | | | | | 8.1±0.9 | 10.6 | 104.2 |
| 15.6 | 15.0±1.6 | 10.4 | 96.3 | | | | 15.4±0.9 | 6.1 | 98.8 |
| 31.3 | 31.6±5.6 | 17.7 | 100.8 | 26.9±6.7 | 24.9 | 86.1 | 30.2±2.1 | 6.8 | 96.4 |
| 62.5 | 61.7±4.6 | 7.4 | 98.7 | 67.4±5.1 | 7.6 | 107.8 | 62.9±1.8 | 2.9 | 100.6 |
| 125.0 | 130.9±15.4 | 11.8 | 104.7 | 130.6±5.7 | 4.4 | 104.5 | 126.7±1.3 | 1.0 | 101.4 |
| 250.0 | 252.1±6.9 | 2.7 | 100.9 | 264.1±16.9 | 6.4 | 105.6 | 259.1±10.3 | 3.9 | 103.7 |
| 500.0 | 493.1±39.2 | 7.9 | 98.6 | 479.7±21.7 | 4.5 | 95.9 | 489.9±14.5 | 2.9 | 97.9 |

| | **M1** | | | **M2** | | | **M3** | | |
|---|---|---|---|---|---|---|---|---|---|
| | Found (nM) | RSD (%) | Accuracy (%) | Found (nM) | RSD (%) | Accuracy (%) | Found (nM) | RSD (%) | Accuracy (%) |
| 0.5 | | | | 0.5±0.1 | 22.3 | 94.0 | | | |
| 1.0 | | | | 1.1±0.1 | 7.5 | 109.7 | 1.0±0.0 | 4.6 | 101.7 |
| 2.0 | 2.0±0.2 | 9.2 | 98.2 | 2.0±0.2 | 11.9 | 100.4 | 2.1±0.4 | 16.9 | 106.2 |
| 3.9 | 3.9±0.6 | 14.7 | 98.9 | 4.1±0.2 | 4.6 | 104.6 | 4.1±0.2 | 4.8 | 105.3 |
| 7.8 | 8.2±0.7 | 9.0 | 104.5 | 8.3±0.4 | 5.0 | 106.2 | 8.4±0.2 | 2.3 | 107.6 |
| 15.6 | 16.7±0.5 | 2.7 | 107.2 | 16.6±1.4 | 8.1 | 106.7 | 17.1±1.5 | 8.9 | 109.6 |
| 31.3 | 33.4±2.1 | 6.4 | 106.8 | 30.0±1.8 | 5.9 | 95.9 | 30.5±1.2 | 4.1 | 97.4 |
| 62.5 | 61.3±3.4 | 5.5 | 98.2 | 62.3±1.6 | 2.6 | 99.6 | 62.0±3.7 | 6.0 | 99.1 |
| 125.0 | 125.1±2.8 | 2.2 | 100.1 | 113.6±2.1 | 1.8 | 90.9 | 109.1±3.2 | 2.9 | 87.3 |
| 250.0 | 232.7±5.8 | 2.5 | 93.1 | 230.1±2.4 | 1.0 | 92.1 | 223.6±8.9 | 4.0 | 89.4 |
| 500.0 | 465.4±9.3 | 2.0 | 93.1 | | | | | | |

Data are means of n=3 analyses.

***Table S5**. The concentration of **1** and **M1 – M3** in human plasma after one dose of roasted coffee brew. Coffee compounds **2** and **3** were not detected in the samples. Note that none of the compounds were detected in any of the plasma samples from the control trial (data not shown).*

| Analyte | Time (h) | eogo | cbdu | wbqj | wbvs | mgjj | ayia | ggot | imhz | rrmk | xrwz | mean±SD (n [c]) |
|---|---|---|---|---|---|---|---|---|---|---|---|---|
| | | Plasma concentration (nM) [a] | | | | | | | | | | |
| 1 | 0 | | | | | | | | | | | |
| | 1 | 43.4 | 31.6 | 49.1 | 36.1 | 34.7 | 36.5 | 49.2 | 70.5 | 42.7 | 26.0 | 41.9±12.5 (10) |
| | 4 | | | 59.7 | 35.2 | 17.7 | | 13.8 [b] | 19.6 | 68.1 | | 40.1±22.9 (5) |
| | 10 | 8.0 [b] | 19.0 | 4.7 [b] | 17.0 | 16.5 | 25.6 | 2.3 [b] | 43.7 | 17.9 | 10.4 [b] | 23.3±10.5 (6) |
| M1 | 0 | | | | | | | | | | | |
| | 1 | 30.0 | 24.6 | 27.5 | 23.8 | 23.6 | 16.4 | 32.2 | 30.6 | 22.0 | 20.2 | 25.1±4.9 (10) |
| | 4 | 4.6 | 2.9 | 36.0 | 17.8 | 12.4 | 1.7 [b] | 9.7 | 10.5 | 19.7 | 1.7 [b] | 11.7±10.7 (8) |
| | 10 | 12.3 | 17.8 | 8.5 | 9.7 | 17.5 | 17.2 | 1.7 [b] | 21.5 | 14.4 | 10.5 | 13.1±5.8 (9) |
| M2 | 0 | | | | | | | | | 0.1 [b] | | |
| | 1 | | 0.1 [b] | 0.01 [b] | | | 0.5 | 0.7 | | | | 0.6±0.1 (2) |
| | 4 | | | | | | 0.1 [b] | | | | | |
| | 10 | 3.1 | 0.9 | 2.8 | 5.1 | 0.8 | 5.6 | 0.6 | 1.1 | | | 2.5±1.9 (8) |
| M3 | 0 | 0.4 [b] | | | 0.9 [b] | | | | | | | |
| | 1 | 0.3 [b] | | | 1.7 | | | | | | | 1.7 (1) |
| | 4 | 0.1 [b] | | 0.9 [b] | 1.9 | | | 2.9 | | 0.7 [b] | | 1.3±1.1 (2) |
| | 10 | 40.8 | 11.6 | 64.1 | 111.0 | 24.3 | | 180.0 | 9.3 | 50.6 | 4.1 | 55.0±57.7 (9) |

[a] empty cells: no compound detected; [b] concentration below LloQ; [c] concentrations above LloQ

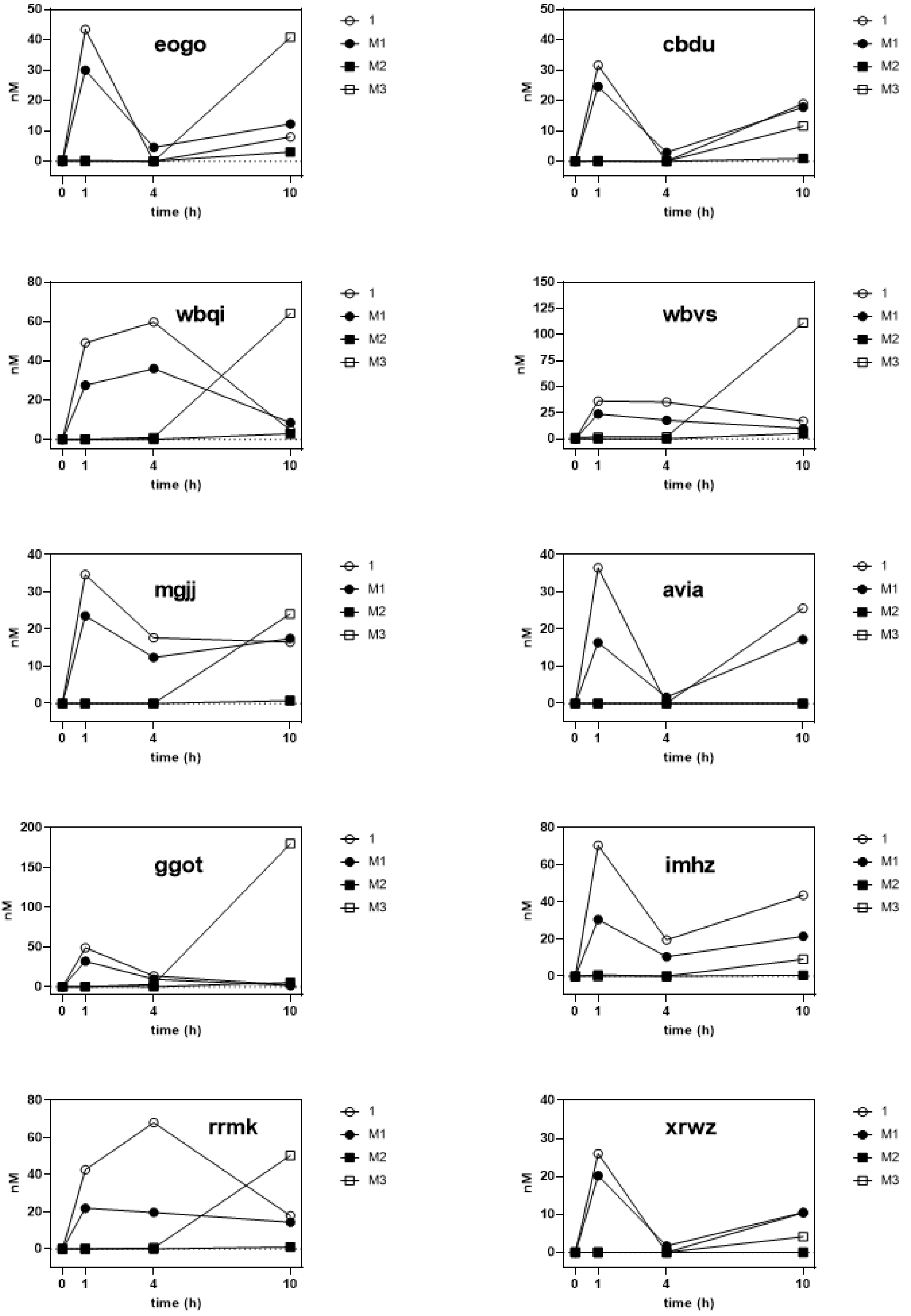


***Figure S3***. *Individual concentration-time plots of the study participants after coffee intervention (concentration <LloQ were substituted with zero).*

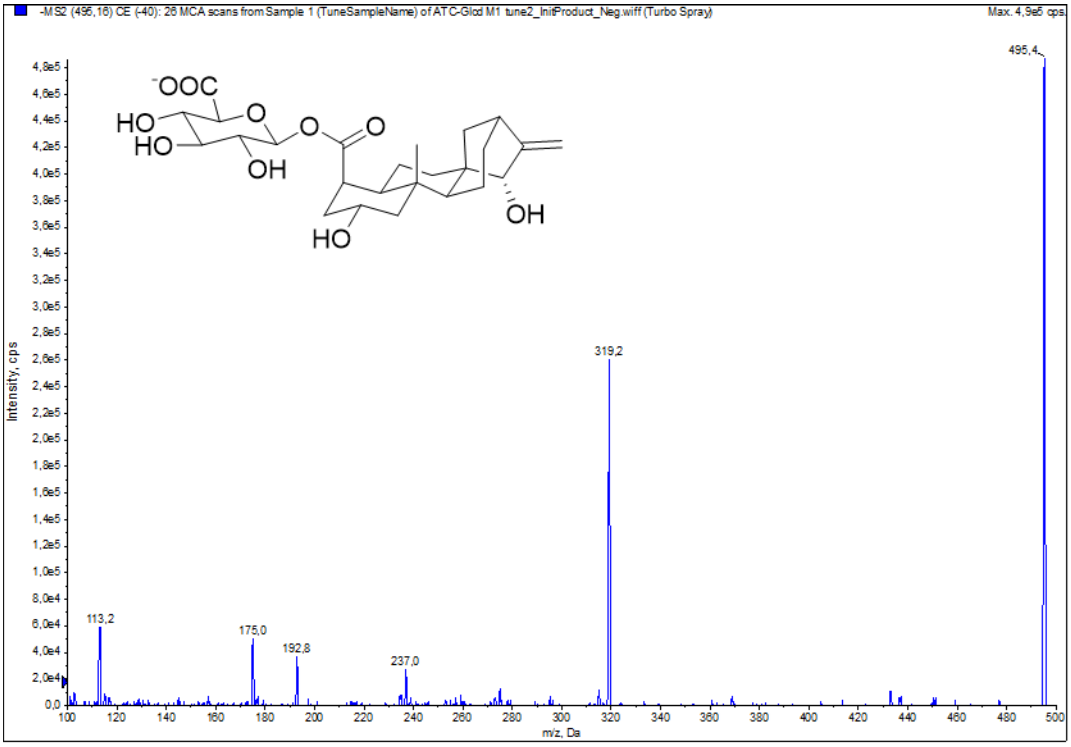


***Figure S4***. *Fragment spectra of M1 (generated during compound tuning in ESI$^-$).*

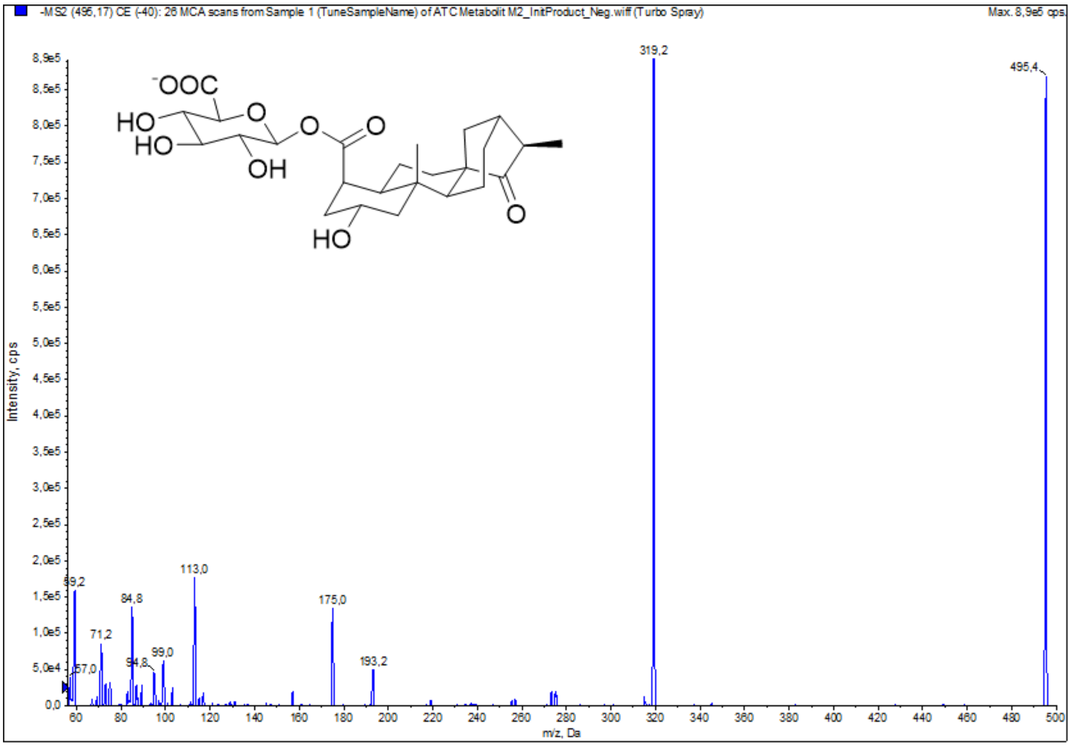


***Figure S5***. *Fragment spectra of M2 (generated during compound tuning in ESI$^-$).*

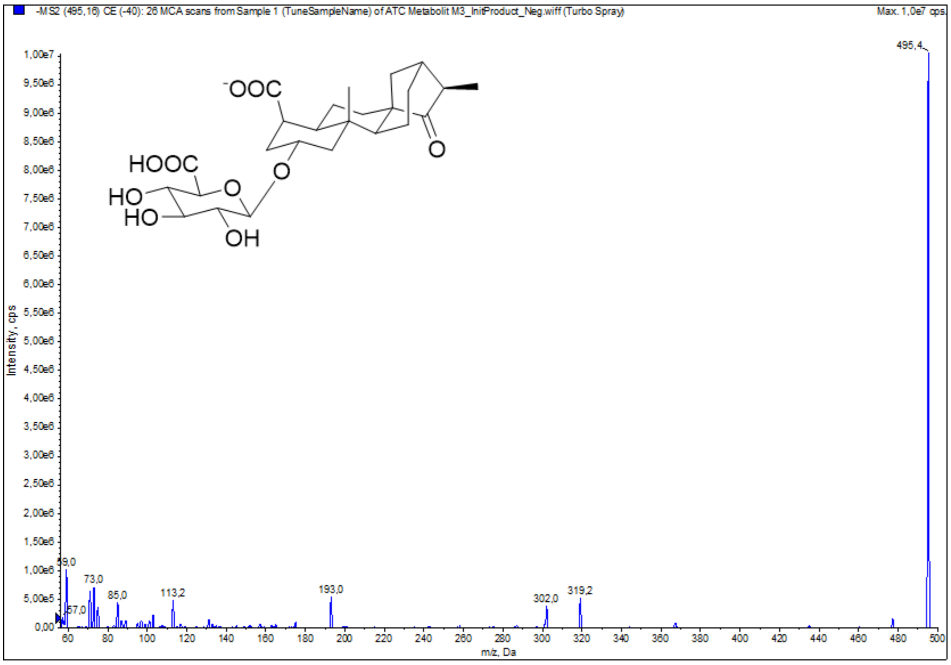


***Figure S6***. *Fragment spectra of M3 (generated during compound tuning in ESI⁻).*

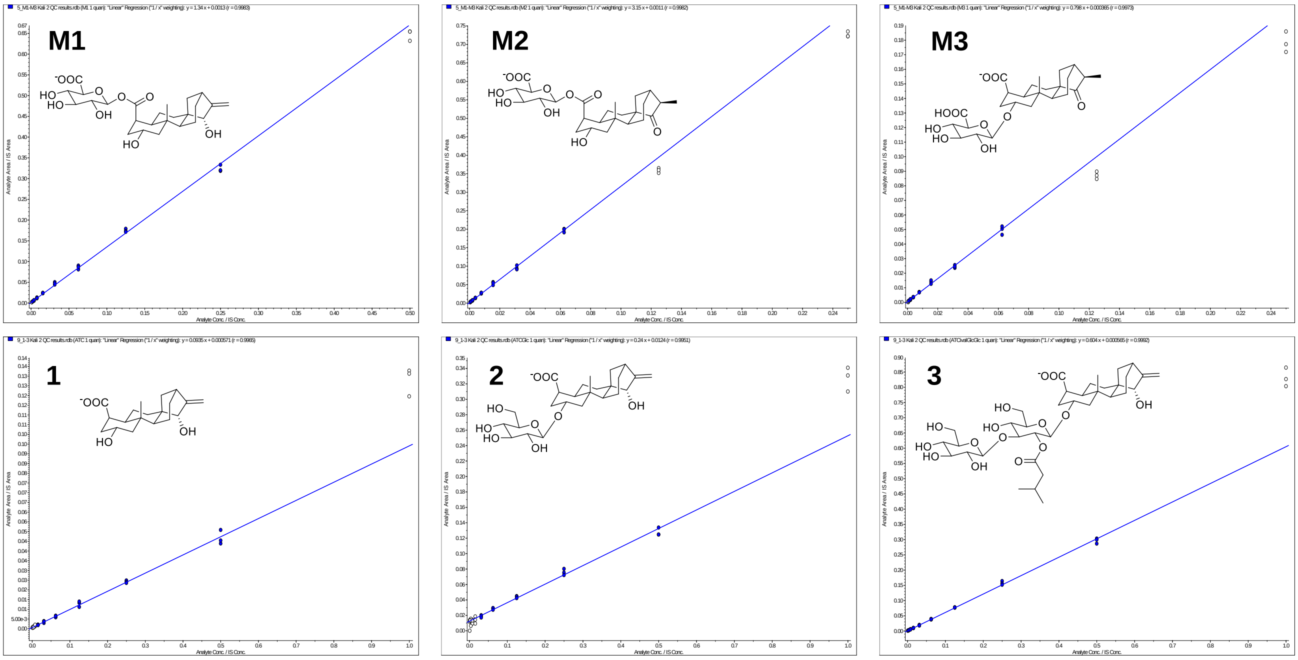


***Figure S7***. *Calibration curves of **1** – **3** and **M1** – **M3** in blank plasma. **1**: m/z 319.01/275.2, 15.6 – 500.0 nM; **2**: m/z 481.18/59.1, 31.3 – 500.0; **3**: m/z 727.26/643.3, 2.0 – 500.0 nM; **M1**: m/z 495.1/319.2, 1.0 – 250 nM; **M2**: m/z 495.17/319.2. 0.5 – 62.5 nM; **M3**: 495.16/192.9, 1.0 – 62.5 nM.*
